# A sequence conserved between CD5 and CD6 binds an FERM domain and exerts a restraint on T‐cell activation

**DOI:** 10.1111/imm.13025

**Published:** 2018-12-10

**Authors:** Johannes Breuning, Marion H. Brown

**Affiliations:** ^1^ Sir William Dunn School of Pathology University of Oxford Oxford UK; ^2^ Present address: GlaxoSmithKline Stevenage UK

**Keywords:** inhibitory/activating receptors, signal transduction, T cell

## Abstract

CD5 and CD6 are related surface receptors that limit and promote T‐cell responses. Co‐stimulatory effects of CD6 depend on binding a cell surface ligand, CD166, and recruitment of the intracellular adaptor proteins GADS and SLP‐76 by C‐terminal phosphotyrosines. We have continued to identify interactions of CD5 and CD6 to understand their roles in T‐cell activation. In a screen to identify binding partners for peptides containing a cytoplasmic sequence, SDSDY conserved between CD5 and CD6, we identified ezrin radixin moesin (ERM) proteins, which link plasma membrane proteins to actin. Purified radixin FERM domain bound directly to CD5 and CD6 SDSDY peptides in a phosphorylation‐dependent manner (K_D_ = 0·5‐2 μm) at 37°. In human T‐cell blasts, mutation of the CD6 SDSDY sequence enhanced CD69 expression in response to CD3 monoclonal antibody. In this proximal readout, interactions of the SDSDY sequence were dominant compared with the C‐terminal tyrosines of CD6. In contrast, in a more downstream readout, interleukin‐2 expression, in response to immobilized CD3 and CD6 monoclonal antibodies, the C‐terminal tyrosines were dominant. The data suggest that varying functional effects of CD6 and potentially CD5 depend on interactions of different cytoplasmic regions with the cytoskeleton and alter depending on the stimuli.

## Introduction

CD5 and CD6 are T‐cell surface receptors that regulate responses to antigen. The two receptors are related, both containing three scavenger receptor cysteine‐rich (SRCR) domains in their extracellular regions and cytoplasmic tails with signalling potential. They have roles in limiting and promoting T‐cell responses *in vitro* and *in vivo*.^1^, ^2^, ^3^, ^4^, ^5^ Progress has been made in identifying molecular interactions that regulate activating effects of CD6. Co‐stimulatory effects of CD6 depend on binding to a cell surface ligand, CD166, and on recruitment of the intracellular adaptor proteins SLP‐76 and GADS.^6^, ^7^ Characterization of genetically manipulated mice lacking CD5 or CD6 revealed that both these receptors could exert inhibitory effects on T‐cell activation.^3^, ^5^ However, challenge of CD5‐ or CD6‐deficient mice resulted in attenuation of autoimmune disease, implicating these receptors in promoting responses.^8^, ^9^ In a different mouse background in a model with the less severe disease, lack of CD6 exacerbated disease.^3^ At the molecular level and in the whole organism, there seems to be a general trend for CD6 that with reduced stimulus, inhibition prevails.^2^, ^3^, ^6^, ^10^ CD5 and CD6 appear to have the potential to mediate activating and inhibitory effects.

Parallels between the behaviour of the two related receptors suggest mechanisms in common. There is a conserved sequence, SDSDY in the cytoplasmic tails of CD5 and CD6. In CD5, there is *in vitro* and *in vivo* evidence for activating and inhibitory functions of the SDSDY sequence.^11^, ^12^ In genetically manipulated mice, knocked‐in CD5 with a deletion in the SDSDY sequence had a similar effect in reducing autoimmune disease compared with CD5‐deficient cells, indicating that the SDSDY sequence is critical for CD5‐mediated regulation.^11^ In *in vitro* analyses, the effects of mutant CD5 were inhibitory or activating depending on the stimulus and assay.^11^ Mapping sites for serine phosphorylation of CD5 and CD6 showed that the SDSDY sequence can be phosphorylated and is a substrate for casein kinase II.^13^, ^14^ Phosphorylated forms of both serines and tyrosine residues in the SDSDY sequence in native CD5 in activated cells have been identified in mass spectrometry screens.^15^

We report interactions of ezrin, radixin and moesin (ERM) proteins with the SDSDY sequence of CD5 and CD6, identified using a proteomics approach. We measured a direct interaction between a FERM domain of an ERM protein and CD5 and CD6 peptides using surface plasmon resonance. The affinities of the interactions were dependent on phosphorylation of the SDSDY sequence. In cells expressing recombinant receptors, mutation of the SDSDY sequence to prevent phosphorylation altered responses. An inhibitory effect of the CD6 SDSDY sequence on proximal signalling in primary CD4^+^ T cells was observed whereas the activating effect of C‐terminal phosphotyrosines, which bind GADS and SLP‐76, dominated in a more downstream readout.

## Material and methods

### Peptides, pulldown and mass spectrometry

Peptides representing human sequences and biotinylated at the N terminus were obtained from Peptide Protein Research Ltd (Hampshire, UK): CD5 pSDpSDpY, PDNSpSDpSDpYDLHGAQRL; CD5 SDSDY, PDNSSDSDYDLHGAQRL, CD6 pSDpSDpY, GpSDpSDpYEHYDFSAQ; CD6 SDSDY: GSDSDYEHYDFSAQ; control peptide (CD5), HVDNEYSQPPRNS; ICAM‐2 (NP_001093259 FERM binding site (amino acids 251–275): HLRQQRMGTYGVRAAWRRLPQAFRP.^16^

Pulldowns and mass spectrometry have been described elsewhere.^6^ Briefly, peptides CD5 pSDpSDpY, CD6 pSDpSDpY or a control peptide derived from a region around a more proximal cytoplasmic tyrosine of CD5 were immobilized on magnetic beads and used to isolate interacting proteins from Jurkat T‐cell lysates. Interacting proteins were processed, then digested on a filter, and the peptides were loaded onto the Ultimate 300 RSLCnano high‐performance liquid chromatography (Dionex, Sunnyvale, CA) ‐tandem mass spectrometry Q Exactive Orbitrap machine using a 25 cm by 75 μm inner diameter picotip analytical column (New Objective, Woburn, MA) in the central proteomics facilities at the Sir William Dunn School of Pathology, University of Oxford. Spectra analysis was conducted using the mascot search engine.^17^

### Soluble recombinant proteins and surface plasmon resonance

Recombinant GST‐tagged FERM domain of radixin constructed from mouse cDNA encoding amino acid residues 1–310, which are 100% identical between mouse and human in the pET49b(+) vector was kindly provided by Toshio Hakoshima (Nara Institute of Science and Technology, Japan).^16^ Radixin FERM was expressed in Rosetta (DE3) pLysS competent cells (Novagen, Madison, WI) and affinity purified using Glutathione Sepharose 4B (GE Healthcare, Chicago, IL). The GST tag was cleaved with HRV 3C protease (Novagen). Cleaved radixin FERM domains were then further purified using size exclusion chromatography.

For surface plasmon resonance analysis, CD5 pSDpSDpY, CD5 SDSDY, CD6 pSDpSDpY and CD6 SDSDY and ICAM‐2 peptides were immobilized on streptavidin‐coated CM5 chips in a BIAcore 3000 machine. Radixin FERM was injected over the immobilized peptides at 37°. Background signals of radixin FERM over a streptavidin‐coated flow cell were subtracted and equilibrium binding data were fitted using a 1 : 1 Langmuir binding model using graphpad prism 7 software.

### Expression of recombinant CD6

Chimeric CD6 (Genbank: HSU34623) containing rat CD6 domain 1 and cytoplasmic mutants were expressed as fusion proteins with enhanced green fluorescent protein (EGFP) using lentiviral vector (pHR‐EGFP) details and detected with rat CD6 domain 1 (OX52) and human CD6 domain 3 (OX124) monoclonal antibodies (mAbs) as described elsewhere.^6^

### Cellular assays

Primary T‐cell assays were conducted as described previously.^6^ Briefly, primary CD4^+^ T cells were isolated using RosetteSep™ and transduced with lentivirus. Cells were stimulated for 18 hr at 37° with platebound CD3 (UCHT1) at a range of concentrations and/or platebound rat CD6 domain 1 mAbs (OX52; 1–5 μg/ml) for CD69 expression and for interleukin‐2 (IL‐2) expression, with immobilized CD3 (2 μg/ml), rat CD6 domain 1 (OX52; 5 μg/ml) or a combination of CD3 and OX52 immobilized together at 5 μg/ml.^6^ CD69 and IL‐2 expression were measured (percentage positive cells) by flow cytometry, gating on EGFP‐positive cells. Experiments were performed once or twice for each donor with duplicate samples for CD69 and once with duplicate or single samples for IL‐2. Data from donors for CD69 and IL‐2, respectively: untransduced *n* = 11, *n* = 14; wild‐type *n* = 11, *n* = 14; SDSDF *n* = 6, *n* = 10; ADADY *n* = 6, *n* = 10; ADADF *n* = 10, *n* = 14; Y629F Y662F *n* = 11, *n* = 13; ADADY Y629F Y662F *n* = 5, *n* = 8; ADADF Y629F Y662F *n* = 5, *n* = 6 were combined. Statistical analyses using analysis of variance with correction for multiple comparisons (Sidak) were carried out using graphpad prism 7 software.

## Results

### CD5 and CD6 pSDpSDpY peptides isolated ERM proteins

To identify potential interacting binding partners for the CD5 and CD6 cytoplasmic SDSDY sequence, we conducted pulldown experiments using Jurkat cell lysates and CD5 or CD6 serine and tyrosine‐phosphorylated pSDpSDpY peptides. Five proteins were repeatedly isolated with CD5 and CD6 pSDpSDpY peptides and were not identified in pulldowns with an unrelated control peptide (Table 1). There was no enrichment for SH2 or PTB domain‐containing proteins by the phosphorylated peptides. Proteins isolated specifically by the pSDpSDpY peptides include the 4·1, (FERM) domain‐containing ERM proteins and the Na^+^/H^+^ exchange regulatory cofactor (NHE‐RF) 1 and 2. ERM proteins have an established role in connecting plasma membrane proteins to the underlying actin cytoskeleton.^18^ NHE‐RF is a well‐established ERM protein binding partner.^18^ These pulldown results suggest that CD5 and CD6 pSDpSDpY sequences associate with ERM proteins or possibly NHE‐RF but do not give an indication which of these proteins bind directly or if they are part of a larger protein complex.

**Table 1:** CD5 and CD6 pSDpSDpY peptides isolated ERM proteins from Jurkat T‐cell lysates

Protein	Unique peptides^a^		% sequence coverage^d^	
	CD5^b^	CD6^c^	CD5	CD6
Ezrin	24–27	9	42–46	24
Moesin	25–35	10	49–45	32
Radixin	7–13	0	25–31	0
NHE‐RF^1^	7–13	0	25–50	0
NHE‐RF^2^	6–8	2	18–33	8

a Peptides only found in the target protein, each number represents one independent experiment.

b Peptides found in three experiments.

c Peptides found in one experiment.

d Protein sequence covered by the identified peptides.

### The radixin FERM domain directly binds to CD5 and CD6 pSDpSDpY

It was plausible that the cytoplasmic regions of CD5 and CD6 bound directly to FERM domains of the ERM proteins in a manner comparable with several other transmembrane proteins.^16^ We tested for direct binding of CD5 and CD6 to FERM domains using surface plasmon resonance. We chose a peptide from the transmembrane protein ICAM‐2 as a positive control.^16^ The finding that ezrin and moesin were most consistently identified as associated with the CD5 and CD6 peptides is likely to be because they are the major ERM proteins expressed in T cells.^19^ As FERM domains of ERM proteins are highly conserved and we were not successful in producing high‐quality monomeric moesin FERM domain, we used the radixin FERM domain to test for direct binding to CD5 and CD6.

**Figure 1:**
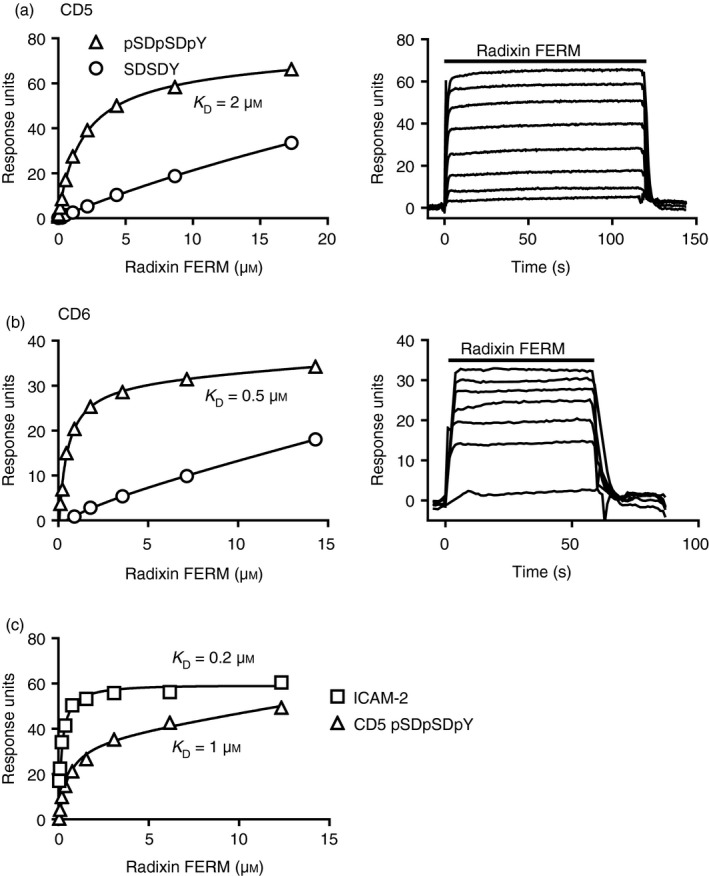
CD5 and CD6 pSDpSDpY peptides bind directly to the FERM domain of radixin. Equilibrium binding fitted curves and K_D_s (left) were derived from the sensogram data (right and not shown) for twofold serial dilutions of the radixin FERM domain injected over peptides: CD5 (a, c) and CD6 (b) pSDpSDpY and unphosphorylated SDSDY and ICAM‐2 (c). Representative data of three independent experiments for CD5 and CD6 are shown.

Recombinant monomeric radixin FERM was injected over CD5 or CD6 pSDpSDpY or non‐phosphorylated SDSDY peptides or a peptide derived from a known FERM domain binding site in ICAM‐2 at 37°. Representative traces for radixin FERM domain over CD5 and CD6 phosphorylated peptides are typical for low‐affinity equilibrium binding by monomeric protein (Fig. 1, right panels). Dissociation constants were measured by equilibrium binding, which is not affected by mass transport, which limits analysis of rapid kinetics.^20^ Fitting equilibrium binding data for three experiments for binding of radixin FERM domain to the CD5 and CD6 phosphorylated pSDpSDpY‐containing peptides gave a K_D_ = 1–2 μm for CD5 and K_D_ = 0·5–2 μm for CD6. The affinity of radixin FERM for the ICAM‐2 peptide was measured as K_D_ ≥ 0·2 μm, indicating that the radixin FERM domain protein was active (Fig. 1c). A higher K_D_ compared with previous measurements for binding of radixin FERM domain to a mouse ICAM‐2 peptide may be explained by differences in temperature and peptide sequence.^16^

Binding of radixin FERM domain to CD5 and CD6 non‐phosphorylated SDSDY peptides was weak and did not reach saturation (Fig. 1a, b). These data indicate that not only are the interactions between CD5 and CD6 and the ERM proteins direct but they are dependent on phosphorylation of the SDSDY sequence.

### The CD6 SDSDY sequence inhibits proximal T‐cell activation

**Figure 2:**
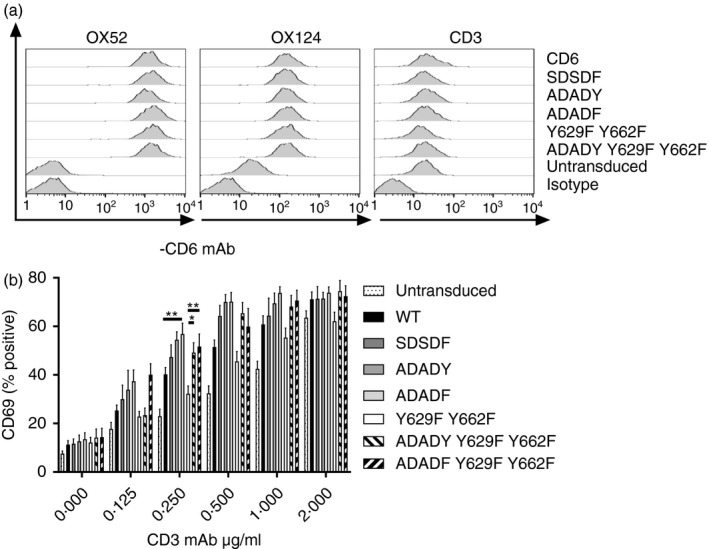
CD6 SDSDY interactions restrain T‐cell activation. (a) Flow cytometry analysis of primary human CD4^+^ T cells transduced with human CD6 with the SRCR domain swapped with the rat CD6 SRCR domain 1 or mutants and stained with rat domain 1 CD6 (OX52), total CD6 (human CD6 domain 3, OX124), CD3 and isotype control monoclonal antibodies (mAbs). (b) T cells were stimulated with platebound CD3 mAb for 18 hr and then CD69 expression was measured by flow cytometry. Means ± SEM from 5 to 11 donors are shown. Statistical analysis revealed differences between cells expressing CD6 versus ADADF (*P* < 0·01) and Y629F Y662F versus ADADY Y629F Y662F (*P* < 0·05) or versus ADADF Y629F Y662F (*P* < 0·01).

We tested the importance of phosphorylation of the SDSDY sequence for CD6‐mediated signalling in primary human CD4^+^ cells. T‐cell blasts were transduced with CD6 containing rat CD6 domain 1; wild‐type cytoplasmic region (CD6), a single tyrosine mutant (SDSDF), a double serine mutant (ADADY), a triple mutant (ADADF), the C‐terminal mutant (Y629F Y662F) or with both regions mutated, (ADADY Y629F Y662F or ADADF Y629F Y662F) as EGFP fusion proteins.^6^ All recombinant CD6 proteins were expressed at similar levels measured by rat CD6 domain 1 mAb (OX52) staining (Fig. 2a, left panel and data not shown). The relative increase in CD6 expression was revealed with a human CD6 domain 3 mAb (OX124) (Fig. 2a, middle panel). Transduction of wild‐type or mutant CD6 did not affect the expression of CD3 (Fig. 2a, right panel).

Cells were stimulated with platebound CD3 mAb.^6^ A consistent pattern was observed, and data from several donors (*n* = 5 to *n* = 11) are collated in Fig. 2(b). Differences among CD6 and mutants were most clearly detected at intermediate concentrations of CD3 mAb. Mutation of the residues, which could be phosphorylated in the SDSDY sequence, showed a trend toward increased responses reaching statistical significance in response to CD3 mAb (shown for 0·25 μg/ml) when all three were mutated (Fig. 2b, ADADF). These data indicated that the phosphorylation of the SDSDY sequence regulates CD6 signalling in a manner that differs from the C‐terminal tyrosine motifs (Fig. 2b^6^). The effect of the SDSDY sequence was diminished and became insignificant when the cells were stimulated with CD3 and CD6 mAbs (data not shown).

### The CD6 SDSDY sequence attenuates proximal T‐cell activation

We assessed the relative effects of the two regions of the cytoplasmic region of CD6 on proximal T‐cell receptor‐mediated signalling by combining mutations of the SDSDY and the C‐terminus (ADADY Y629F Y662F and ADADF Y629F Y662F). In the proximal signalling readout of CD69 expression, responses by ADADY Y629F Y662F in which just the two serines in the SDSDY sequence were mutated and ADADF Y629F Y662F matched those of the SDSDY mutants, not the C‐terminal mutant (Y629F Y662F). The responses of ADADY Y629F Y662F and ADAF Y629F Y662F were significantly (shown for CD3 mAb 0·25 μg/ml) raised above that of Y629F Y662F (Fig. 2b). The SDSDY region appeared dominant, and this was observed in the absence of ligation of the extracellular region of CD6 in these assays.

### Interactions of the CD6 C terminus compared with SDSDY are dominant in a downstream readout of T‐cell activation

**Figure 3:**
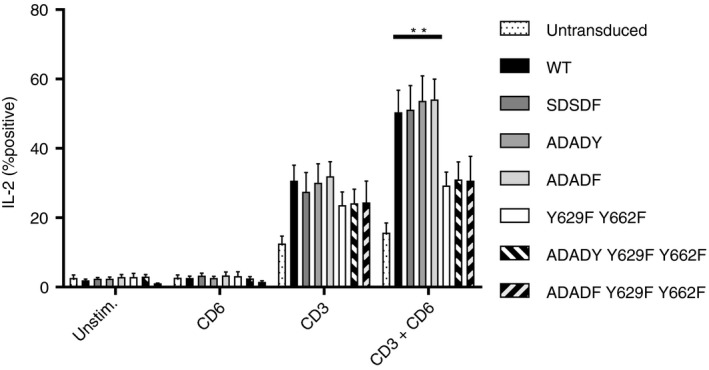
CD6 Y629 Y662 interactions dominate downstream in T‐cell activation. T cells were stimulated with platebound CD3, rat CD6 domain 1 or CD3 and rat CD6 domain 1 monoclonal antibodies (mAbs) for 18 hr, and then, interleukin‐2 (IL‐2) expression was measured by flow cytometry. Means ± SEM from 6 to 14 donors are shown. Statistical analysis revealed differences between cells stimulated with CD3^+^ CD6 mAbs expressing CD6 versus Y629F Y662F (*P* < 0·01) but not between Y629F Y662F versus ADADY Y629F Y662F or versus ADADF Y629F Y662F.

The analysis of the effects of mutating CD6 on CD69 expression indicated that the SDSDY sequence can exert a restraint on proximal events in T‐cell activation. To assess the relative effects of the SDSDY sequence and the C terminus of CD6 on a downstream measure of activation, we analysed IL‐2 production in a single cell assay. Figure 3 shows combined data of the means from donors (*n* = 6 to *n* = 14). As we have shown previously, expression of CD6 increased the percentage of IL‐2‐positive cells in response to CD3 mAb and more markedly in the presence of the rat CD6 domain 1 mAb (Fig. 3, 6). Stimulation with the combination of CD3 and CD6 mAbs revealed a reduced percentage of IL‐2‐positive cells expressing the C‐terminal mutant, Y629F Y662F of CD6 compared with transduced wild‐type CD6 (Fig. 3, 6). The percentage of IL‐2‐positive cells remained reduced in cells expressing ADADY Y629F Y662F or ADADF Y629F Y662F (Fig. 3). The C‐terminal mutations were dominant in a downstream readout when the T‐cell receptor and the extracellular region of CD6 were ligated.

## Discussion

Regulation of T‐cell activation by non‐catalytic tyrosine‐phosphorylated receptors is generally mediated by phosphorylation‐dependent interactions of tyrosine residues and direct recruitment of SH2 domain‐containing proteins, which lead to activating or inhibitory signalling.^21^ Identification of physiologically relevant specific SH2 domain binding partners for the C‐terminal region of these receptors using highly multivalent immobilized peptides has been successful.^6^, ^10^, ^22^ In a reciprocal manner, a complementary approach based on preservation of protein complexes associated with intracellular proteins also identified the interaction between SLP‐76 and CD6.^23^ This latter approach identified CD5 together with SH2 domain‐containing proteins in protein complexes.^24^ However, using the multivalent CD5 and CD6 peptides, we did not isolate a specific SH2 domain binding partner for the SDSDY sequence in CD5 or CD6. Identification of ERM proteins as binding partners for a phosphorylated SDSDY sequence in common between CD5 and CD6 suggests a phosphorylation‐dependent mechanism that regulates the cytoskeleton and is relevant for regulating the activating and inhibitory effects by these receptors.

Binding of the cytoplasmic sequences in CD5 and CD6 to ERM proteins was novel in that it was dependent on phosphorylation. Dependence on phosphorylation suggested that negative charge was important for the interaction. This contrasts with basic residues in an ICAM‐2 peptide being important for the affinity of the interaction.^16^ Transmembrane protein cytoplasmic regions containing less basic and more acidic residues have a reduced affinity generally by as much as ~50‐fold for the radixin FERM compared with ICAM‐2 in measurements made at 25°.^16^ Compared with ICAM‐2, CD5 and CD6 bound to the radixin FERM domain with only a fivefold to 10‐fold lower affinity at 37°. The affinity (K_D_ = 0·5 μm at 37°) of the interaction between the phosphorylated CD6 SDSDY peptide and the radixin FERM domain is comparable with the phosphorylated C‐terminal tyrosine interaction with the SH2 domain of SLP‐76.^10^ These hierarchies indicate that interactions between CD5 and CD6 and ERM proteins are potentially physiologically significant in terms of ERM binding partners for type I transmembrane proteins.

The presence of NHE‐RF adaptor proteins which bind to a different region of the PTB‐like FERM subdomain among proteins isolated by the CD5 and CD6 peptides is consistent with the same or overlapping binding site for ICAM‐2 and the phosphorylated SDSDY sequence.^18^ On the other hand, it is conceivable that the highly negatively charged pSDpSDpY binds to the same positively charged surface as phospholipids such as IP_3_, leading to ERM protein activation and subsequent binding to other transmembrane proteins.^18^

Detection of phosphorylation of a cluster of serines including the two in the SDSDY sequence in the absence of stimulation is consistent with the functional effects of mutating these serines being observed in the absence of a strong stimulus (Fig. 2b^13^). Phosphorylation of the tyrosine in SDSDY and possibly the tyrosine (Y489) three residues downstream (SDSDYEHY_489_) may contribute to the electrostatic interaction with ERM proteins. Mutation of Y489F resulted in increased phosphorylation of CD6, indicating an inhibitory function and, similarly to the SDSDY sequence, was subservient to the effect of a C‐terminal mutation in IL‐2 production.^10^ Inhibitory effects of mutating the SDSDY sequence in recombinant CD6 were more apparent where the stimulus was limited to a CD3 mAb in the absence of CD6 ligation. In genetically manipulated mice, inhibitory effects of CD6 deficiency were observed in the presence of CD3 mAbs and obscured by the addition of co‐stimulation with a CD28 mAb.^3^

Tipping the balance from inhibitory to co‐stimulatory effects of CD6 seems to depend on the stimulus including engagement of other receptors. Increasing the number of antigen‐presenting cells obscured the inhibitory effects of transduced CD6 in a murine T‐cell hybridoma line.^10^ The effect appeared to be dependent on the antigen‐presenting cells, not antigen dose.^10^ A strong stimulus with substantial up‐regulation of CD166 on antigen‐presenting cells will recruit more CD6 to cell‐contact sites, resulting in activating effects dependent on tyrosine phosphorylation and recruitment of GADS/SLP‐76 complexes.^6^, ^25^ The dominant effect of the C‐terminal interactions compared with the SDSDY sequence on downstream events in T‐cell activation supports the conclusion that recruitment of a GADS/SLP‐76 adaptor complex is the major mechanism underlying ligand‐dependent co‐stimulation by CD6.^6^

The region of ICAM‐2 that binds to FERM domains is membrane proximal in the linear amino acid sequence, and membrane proximal basic residues are commonly found in cytoplasmic regions of transmembrane proteins.^16^ The SDSDY region that binds FERM domains in CD5 and CD6 is more distal; in the CD5 cytoplasmic region at 82 amino acids close to the C terminus (94 amino acids) and in CD6, which has a longer cytoplasmic region (244 amino acids), at 58 amino acids from the membrane. Even more distal are interactions between the C terminus of CD6 and a cytoskeletal protein syntenin or the adaptors GADS and SLP‐76, which are also implicated in regulating the cytoskeleton.^6^, ^26^ A specific mechanism of regulation of the cytoskeleton may define a niche for CD6 and for CD5.

During T‐cell activation, regulation of phosphorylation of ERM proteins facilitates redistribution of transmembrane proteins; for example, ezrin moves transiently to the site of cell contact.^19^ CD6 and CD5 can be detected in immunological synapses.^27^ Recruitment of CD6 to the site of cell contact can be achieved through ligand binding to artificially raised levels of CD166 in the absence of the cytoplasmic region of CD6^25^ but it is likely that it contributes under physiological conditions.^28^ Transport of CD6 was sensitive to disruption of the actin cytoskeleton.^28^ Interactions with ERM proteins and the cytoskeleton are also important for adhesion by the CD6 ligand, CD166.^29^ There is a precedent for CD6 having a role in another ERM protein‐dependent process, migration. Loss of CD6 impairs migration of T cells,^9^ and a CD6 mAb inhibits migration of CD4^+^ T cells across the blood–brain barrier.^30^ In an experimental autoimmune encehalomyelitis model of autoimmune disease, CD4^+^ cells showed reduced infiltration of the spinal cord in transgenic mice expressing CD5 with a deletion, SSDS, overlapping the SDSDY sequence, compared with the wild‐type transgene.^12^ A study exploiting CD6 as a homing receptor shows its signalling effects on cytoskeletal activity and includes microscopy demonstrating a link with the actin cytoskeleton.^31^

Can an interaction with ERM proteins account for dual activating and inhibitory effects of CD6 and by association, CD5? In preliminary experiments, mutation of the SDSDY sequence in CD5 in primary cells resulted in enhanced or reduced CD69 expression depending on the donor (JB and MHB, unpublished observations). In Jurkat cells, mutation of the SDSDY sequence in CD6 revealed that it had an activating effect on CD69 expression (JB and MHB, unpublished observations) leading to speculation that interactions of the SDSDY sequence contribute to the balance between inhibitory and activating functional effects by affecting transport or segregation of receptors rather than a direct interaction with a signalling enzyme.^21^, ^32^, ^33^

## Disclosure

None declared.

## References

[1] Zimmerman AW , Joosten B , Torensma R , Parnes JR , van Leeuwen FN , Figdor CG . Long‐term engagement of CD6 and ALCAM is essential for T‐cell proliferation induced by dendritic cells. Blood 2006; 107:3212–20. 16352806 10.1182/blood-2005-09-3881

[2] Oliveira MI , Goncalves CM , Pinto M *et al* CD6 attenuates early and late signaling events, setting thresholds for T‐cell activation. Eur J Immunol 2012; 42:195–205. 21956609 10.1002/eji.201040528 PMC3298641

[3] Orta‐Mascaro M , Consuegra‐Fernandez M , Carreras E *et al* CD6 modulates thymocyte selection and peripheral T cell homeostasis. J Exp Med 2016; 213:1387–97. 27377588 10.1084/jem.20151785 PMC4986531

[4] Palin AC , Love PE . CD5 helps aspiring regulatory T cells ward off unwelcome cytokine advances. Immunity 2015; 42:395–6. 25786168 10.1016/j.immuni.2015.02.018

[5] Tarakhovsky A , Kanner SB , Hombach J *et al* A role for CD5 in TCR‐mediated signal transduction and thymocyte selection. Science 1995; 269:535–7. 7542801 10.1126/science.7542801

[6] Breuning J , Brown MH . T cell costimulation by CD6 is dependent on bivalent binding of a GADS/SLP‐76 complex. Mol Cell Biol 2017; 37:e00071–17. 28289074 10.1128/MCB.00071-17 PMC5440646

[7] Chappell PE , Garner LI , Yan J *et al* Structures of CD6 and its ligand CD166 give insight into their interaction. Structure 2015; 23:1426–36. 26146185 10.1016/j.str.2015.05.019 PMC4533223

[8] Axtell RC , Webb MS , Barnum SR , Raman C . Cutting edge: critical role for CD5 in experimental autoimmune encephalomyelitis: inhibition of engagement reverses disease in mice. J Immunol 2004; 173:2928–32. 15322150 10.4049/jimmunol.173.5.2928

[9] Li Y , Singer NG , Whitbred J , Bowen MA , Fox DA , Lin F . CD6 as a potential target for treating multiple sclerosis. Proc Natl Acad Sci USA 2017; 114:2687–92. 28209777 10.1073/pnas.1615253114 PMC5347585

[10] Hassan NJ , Simmonds SJ , Clarkson NG *et al* CD6 regulates T‐cell responses through activation‐dependent recruitment of the positive regulator SLP‐76. Mol Cell Biol 2006; 26:6727–38. 16914752 10.1128/MCB.00688-06 PMC1592849

[11] Sestero CM , McGuire DJ , De Sarno P *et al* CD5‐dependent CK2 activation pathway regulates threshold for T cell anergy. J Immunol 2012; 189:2918–30. 22904299 10.4049/jimmunol.1200065 PMC3436980

[12] Axtell RC , Xu L , Barnum SR , Raman C . CD5‐CK2 binding/activation‐deficient mice are resistant to experimental autoimmune encephalomyelitis: protection is associated with diminished populations of IL‐17‐expressing T cells in the central nervous system. J Immunol 2006; 177:8542–9. 17142752 10.4049/jimmunol.177.12.8542 PMC2744950

[13] Bonet L , Farnos M , Martinez‐Florensa M , Martinez VG , Lozano F . Identification of functionally relevant phoshorylatable serine clusters in the cytoplasmic region of the human CD6 lymphocyte surface receptor. FEBS Lett 2013; 587:2205–13. 23711376 10.1016/j.febslet.2013.05.043

[14] Calvo J , Vilda JM , Places L *et al* Human CD5 signaling and constitutive phosphorylation of C‐terminal serine residues by casein kinase II. J Immunol 1998; 161:6022–9. 9834084

[15] Salek M , McGowan S , Trudgian DC *et al* Quantitative phosphoproteome analysis unveils LAT as a modulator of CD3ζ and ZAP‐70 tyrosine phosphorylation. PLoS ONE 2013; 8:e77423. 24204825 10.1371/journal.pone.0077423 PMC3813684

[16] Hamada K , Shimizu T , Yonemura S , Tsukita S , Tsukita S , Hakoshima T . Structural basis of adhesion‐molecule recognition by ERM proteins revealed by the crystal structure of the radixin‐ICAM‐2 complex. EMBO J 2003; 22:502–14. 12554651 10.1093/emboj/cdg039 PMC140724

[17] MacLean B , Eng JK , Beavis RC , McIntosh M . General framework for developing and evaluating database scoring algorithms using the TANDEM search engine. Bioinformatics 2006; 22:2830–2. 16877754 10.1093/bioinformatics/btl379

[18] Fehon RG , McClatchey AI , Bretscher A . Organizing the cell cortex: the role of ERM proteins. Nat Rev Mol Cell Biol 2010; 11:276–87. 20308985 10.1038/nrm2866 PMC2871950

[19] Shaffer MH , Dupree RS , Zhu P *et al* Ezrin and moesin function together to promote T cell activation. J Immunol 2009; 182:1021–32. 19124745 10.4049/jimmunol.182.2.1021 PMC3491660

[20] der van Merwe P . Surface Plasmon Resonance In: Chowdhry SEHBZ, editor. Protein‐ligand interactions: hydrodynamic and colorimetry 2. USA: Oxford University Press; 2001 137–70.

[21] Dushek O , Goyette J , van der Merwe PA . Non‐catalytic tyrosine‐phosphorylated receptors. Immunol Rev 2012; 250:258–76. 23046135 10.1111/imr.12008

[22] Mihrshahi R , Barclay AN , Brown MH . Essential roles for Dok2 and RasGAP in CD200 receptor‐mediated regulation of human myeloid cells. J Immunol 2009; 183:4879–86. 19786546 10.4049/jimmunol.0901531 PMC2788151

[23] Roncagalli R , Hauri S , Fiore F *et al* Quantitative proteomics analysis of signalosome dynamics in primary T cells identifies the surface receptor CD6 as a Lat adaptor‐independent TCR signaling hub. Nat Immunol 2014; 15:384–92. 24584089 10.1038/ni.2843 PMC4037560

[24] Voisinne G , Garcia‐Blesa A , Chaoui K *et al* Co‐recruitment analysis of the CBL and CBLB signalosomes in primary T cells identifies CD5 as a key regulator of TCR‐induced ubiquitylation. Mol Syst Biol 2016; 12:876. 27474268 10.15252/msb.20166837 PMC4965873

[25] Castro MA , Oliveira MI , Nunes RJ *et al* Extracellular isoforms of CD6 generated by alternative splicing regulate targeting of CD6 to the immunological synapse. J Immunol 2007; 178:4351–61. 17371992 10.4049/jimmunol.178.7.4351

[26] Gimferrer I , Ibanez A , Farnos M *et al* The lymphocyte receptor CD6 interacts with syntenin‐1, a scaffolding protein containing PDZ domains. J Immunol 2005; 175:1406–14. 16034076 10.4049/jimmunol.175.3.1406

[27] Gimferrer I , Calvo M , Mittelbrunn M *et al* Relevance of CD6‐mediated interactions in T cell activation and proliferation. J Immunol 2004; 173:2262–70. 15294938 10.4049/jimmunol.173.4.2262

[28] Meddens M , Mennens S , Celikkol F *et al* Biophysical characterization of CD6‐TCR/CD3 interplay in T cells. Front Immunol 2018; 9:2333. in press. 30356797 10.3389/fimmu.2018.02333 PMC6189472

[29] Tudor C , te Riet J , Eich C *et al* Syntenin‐1 and ezrin proteins link activated leukocyte cell adhesion molecule to the actin cytoskeleton. J Biol Chem 2014; 289:13445–60. 24662291 10.1074/jbc.M113.546754 PMC4036352

[30] Cayrol R , Wosik K , Berard JL *et al* Activated leukocyte cell adhesion molecule promotes leukocyte trafficking into the central nervous system. Nat Immunol 2008; 9:137–45. 18157132 10.1038/ni1551

[31] Samaha H , Pignata A , Fousek K *et al* A homing system targets therapeutic T cells to brain cancer. Nature 2018; 561:331–7. 30185905 10.1038/s41586-018-0499-y PMC6402337

[32] Freeman SA , Vega A , Riedl M *et al* Transmembrane pickets connect cyto‐ and pericellular skeletons forming barriers to receptor engagement. Cell 2018; 172:e10. 10.1016/j.cell.2017.12.023 PMC5929997 29328918

[33] Mylvaganam SM , Grinstein S , Freeman SA . Picket‐fences in the plasma membrane: functions in immune cells and phagocytosis. Semin Immunopathol 2018; In press 10.1007/s00281-018-0705-x . [Epub ahead of print] 30209546

